# Supplementary material for: Magia: Robust Automated Image Processing and Kinetic Modeling Toolbox for PET Neuroinformatics
Source: Front Neuroinform. 2020 Feb 4;14:3. doi: 10.3389/fninf.2020.00003 (PMC7012016; doi:10.3389/fninf.2020.00003)
Supplement: Supplementary file 1 [file Table_1.DOCX]

**Supplementary material**

**Supplementary Figure 1.** Differences in volumes of manually and automatically produced reference regions.

**Supplementary Figure 2.** Between-subject average reference region radioactivity distributions. Blue = Magia; red = manual.

**Supplementary Figure 3.** Differences in AUCs of manually and automatically produced reference regions.

**Supplementary Figure 1.** Differences in volumes of manually and automatically produced reference regions.


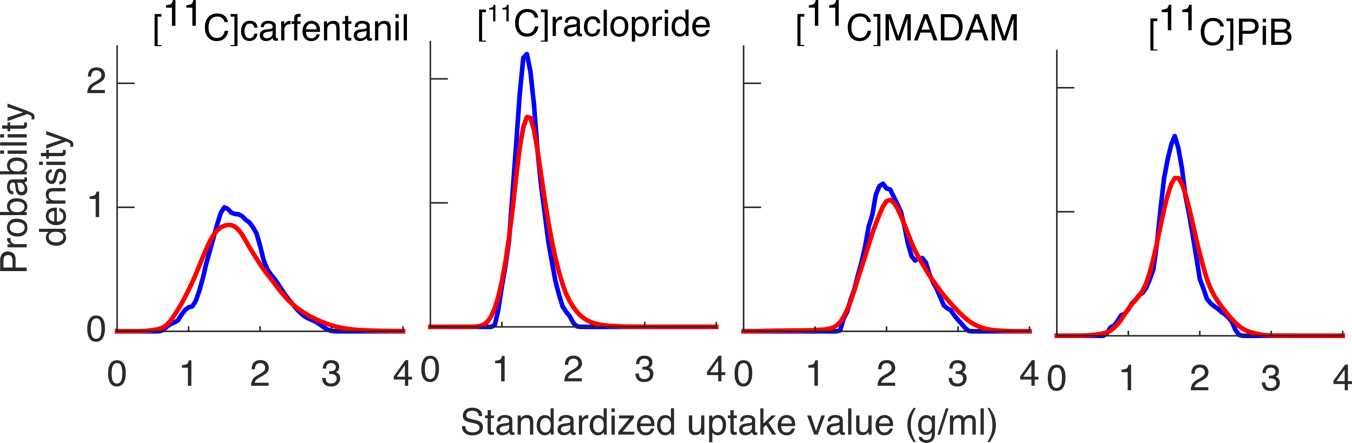


**Supplementary Figure 2.** Between-subject average reference region radioactivity distributions. Blue = Magia; red = manual.


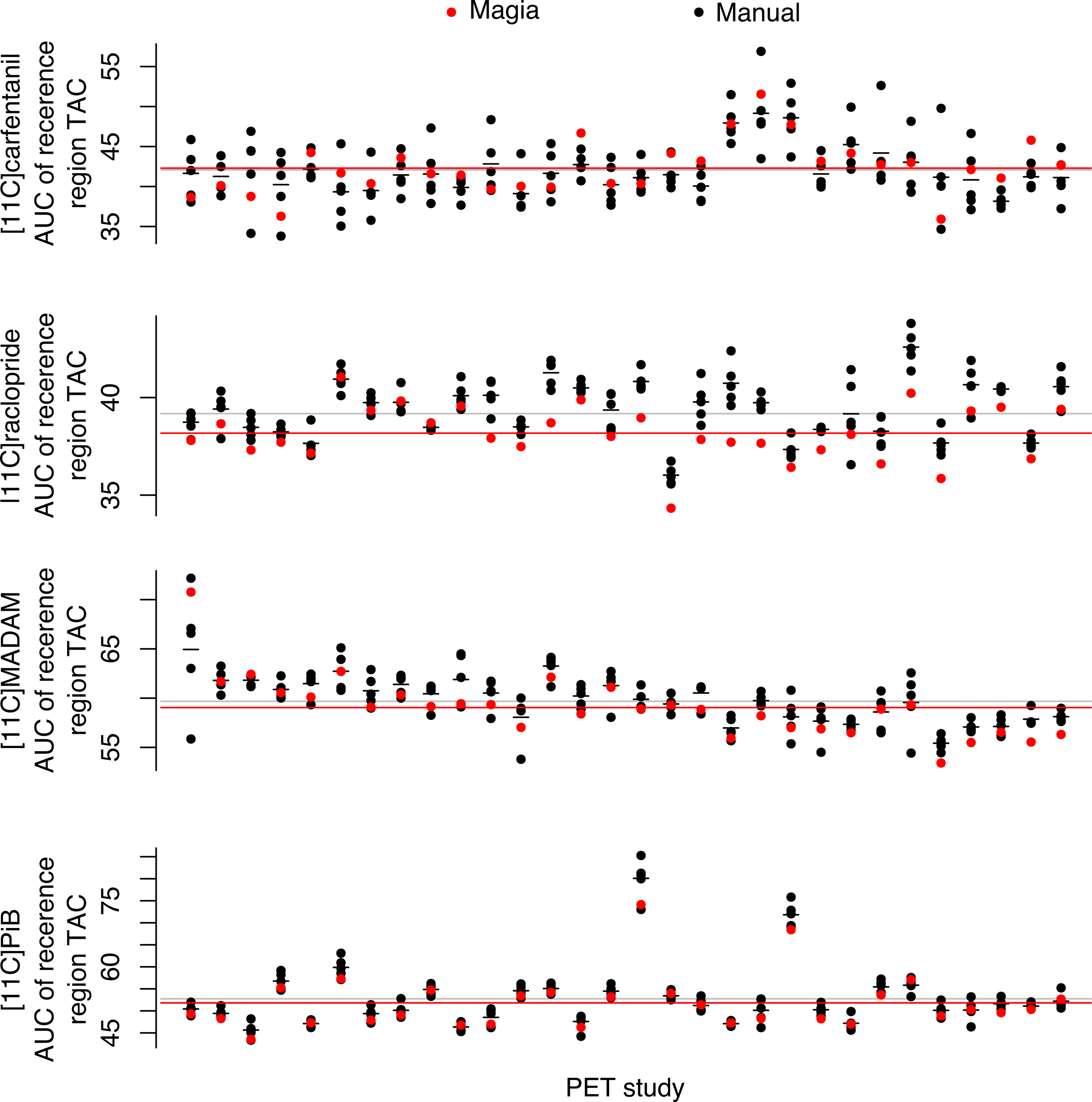


**Supplementary Figure 3.** Differences in AUCs of manually and automatically produced reference regions.
